# Advances and Challenges at the Waste-to-Bioenergy/Biorefinery Nexus

**DOI:** 10.1155/2018/3642363

**Published:** 2018-06-14

**Authors:** Shijian Ge, Joseph Usack, Bin Ma

**Affiliations:** ^1^Jiangsu Key Laboratory of Chemical Pollution Control and Resources Reuse, School of Environmental and Biological Engineering, Nanjing University of Science and Technology, Xiao Ling Wei 200, Nanjing, Jiangsu 210094, China; ^2^University of Tübingen, Center for Applied Geoscience. Hölderlinstr. 12, Tübingen 72074, Germany; ^3^Department of Environmental Sciences and Engineering, Hainan University, Renmin Avenue 58, Haikou, Hainan 570228, China

Population growth and industrialization across the globe are leading to the production of larger waste volumes. This will occur even though the current rate of waste production is already exceeding the capacity of existing waste management infrastructure in many parts of the world. The environmental consequences of inadequate waste management are already being realized on both the small and global scale through, for example, the pollution of local air/water bodies and climate change. While these increases in waste production pose many challenges, they also present many opportunities to develop novel technologies to not only better stabilize or utilize these wastes, but also recover valuable resources, such as energy, green chemicals, and nutrients. Thus, in response to this emerging scientific field, this special issue was organized to serve as a platform for novel research that addresses these challenges and specific applications of the removal, utilization, and bioconversion of biowaste. A total of 9 quality papers were published covering various topics as presented below.

The paper authored by Z. Chen et al. presents a review on determination of microalgal lipid content and fatty acid for biofuel production by summarizing and comparing different approaches of extraction and quantification of microalgal lipids including the pretreatment of microalgal cells, as well as describing the principles and procedures for the production and quantification of fatty acids in detail. Apart from the traditional extraction methods using conventional organic solvents, this review also introduces newly-developed lipid-extraction techniques, such as CO_2_-based solvents, ionic liquids, and switchable solvents. The authors make specific suggestions about the determination methods of microalgal lipids (*i.e.*, gravimetric method, Nile red lipid visualization method, sulfo-phospho-vanillin method, and thin-layer chromatography method) as well.

The paper titled “Effect of Free Nitrous Acid on Nitrous Oxide Production and Denitrifying Phosphorus Removal by Polyphosphorus-Accumulating Organisms in Wastewater Treatment” by Z. Miao et al. studied the relationship between free nitrite acid (FNA) and nitrous oxide (N_2_O) in denitrifying phosphorus removal process. The results showed that FNA, rather than nitrite and pH, was likely the true inhibitor of N_2_O production. Moreover, the nitrite reduction rate, phosphorus uptake rate, N_2_O reduction rate, and PHA degradation rate also decreased as the concentration of FNA increased. The highest proportion of N_2_O to TN was 78.42% because FNA prevented the step from NO_2_-to-N_2_O and N_2_O-to-N_2_. Meanwhile, this part of dissolved N_2_O, as a significant greenhouse gas (~300 times greater warming potential than CO_2_), could be diffused into air.

The paper written by Q. Chang et al. proposed an alternative low- and high-ammonium influent regime to maintain a completely autotrophic nitrogen removal over nitrite (CANON) treatment for low ammonium wastewater. Their findings showed that excessive proliferation of nitrite oxidizing bacteria (NOB) in a low-ammonium environment was still a challenge for stable CANON operation. However, with 28 days of high-ammonium treatment combined with a controlled sludge retention time, the overproliferation of NOB in the low ammonium operational period could be avoided. They suggested that when the nitrite oxidation rate reached 8 g N/m^3^/h, the CANON system should enter the high-ammonium influent operating mode. The proposed strategy can be realized if wastewater treatment plants have a sludge digestion unit, from which the higher-ammonium influent can be supplied.

The paper written by F. Yang et al. employed the Malmquist-Luenberger productivity index to evaluate the productivity change of environmentally friendly production technologies that simultaneously reduce wastewater discharges and generate economic outputs for 30 administrative provinces in China during 2003-2015. During this period, they observed a downward trend and growing spatial disparities for China's water preferable productivity index in many of these provinces. The major cause of these developments can be attributed to environmentally friendly technology changes, while only a minor effect can be attributed to the improvement of the technical efficiency.

The paper authored by S. Xu et al. entitled “Analysis of Bacterial Community Structure of Activated Sludge from Wastewater Treatment Plants in Winter” investigated the microbial-community structure of activated sludge in wastewater treatment plants and identified the bacteria that caused bulking of activated sludge in winter. This result will help optimize wastewater treatment and water reclamation practices.

The paper titled “Efficient Utilization of Waste Carbon Source for Advanced Nitrogen Removal of Landfill Leachate” by K. Wang et al. assessed the nitrogen removal adaptability of a modified single sequencing batch reactor (SBR). The operation mode of the SBR was filling, stirring, aeration, stirring, and settling which could enhance the nitrogen removal rate of leachate. The chemical oxygen demand (COD) and ammonia of the SBR effluent were less than 500 mg/L and 40 mg/L under the condition without a carbon source. Furthermore, the removal rates of COD and total nitrogen were greater than 85% and 95%, respectively. The maximum specific nitrogen removal rate reached 1.48 mg N/h/g VSS. Polyhydroxyalkanoates were the primary carbon source in the sludge for nitrogen removal. In whole experiment period (*i.e., *160 days), the sludge concentration remained nearly unchanged because most of the organic matter in the raw wastewater was used for denitrification.

In the paper titled “Study of Nitrogen Removal Performance When Treating Low Carbon Sewage Using External Solid Carbon Sources in SBBR Systems,” L. Zhang et al. used the waste from corncob processing as the external solid carbon source for biological nitrogen removal. The results showed that a low-nitrogen effluent could be obtained using this wastewater treatment system.

The paper titled “Analysis of the Metabolites of Indole Degraded by an Isolated* Acinetobacter pittii* L1” by Z. Yang et al. isolated an efficient indole degrading* Acinetobacter pittii *L1 from a coking wastewater.* A. pittii *L1 was demonstrated as a promising candidate for the degradation of nitrogen heterocyclic compounds, the production of indigoids, soil remediation, and the treatment of indole containing wastewaters.

In the paper titled “Start-Up and Aeration Strategies for a Completely Autotrophic Nitrogen Removal Process in an SBR,” X. Zhang et al. demonstrated that Planctomycete-like anammox bacteria and* Nitrosomonas*-like aerobic ammonium oxidization bacteria could be cultivated in an SBR using intermittent aeration, so as to achieve autotrophic nitrogen removal for reducing energy consumption of wastewater treatment and water reclamation.

